# From Green Chemistry to Healthy Environments: Silver Nanoparticles as a Dual Antioxidant and Antibacterial Agents for Advancing Biomedicine and Sustainable Wastewater Treatment

**DOI:** 10.3390/bioengineering11121205

**Published:** 2024-11-28

**Authors:** Hamza Moussa, Sarah Hamid, Amal Mameri, Sabrina Lekmine, Hichem Tahraoui, Mohammed Kebir, Nabil Touzout, Farid Dahmoune, Mohammad Shamsul Ola, Jie Zhang, Abdeltif Amrane

**Affiliations:** 1 Laboratory of Management and Valorization of Natural Resources and Quality Assurance (LGVRNAQ), Faculty of Natural and Life Sciences and Earth Sciences, University of Bouira, Bouira 10000, Algeria; h.moussa@univ-bouira.dz (H.M.); mameri@univ-bouira.dz (A.M.); 2 Department of Biology, Faculty of Natural and Life Sciences and Earth Sciences, University of Bouira, Bouira 10000, Algeria; farid.dahmoune@gmail.com; 3 Laboratory of Plant Biotechnology and Ethnobotany, Faculty of Natural and Life Sciences, University of Bejaia, Bejaia 06000, Algeria; sarah.hamid@gmail.com; 4 Biotechnology, Water, Environment and Health Laboratory, Abbes Laghrour University, Khenchela 40000, Algeria; 5 Laboratory of Biomaterials and Transport Phenomena, University of Medea, Medea 26000, Algeria; hichemm.tahraouii@gmail.com (H.T.) nabil12marin.eco@gmail.com (N.T.); 6 Univ Rennes, Ecole Nationale Supérieure de Chimie de Rennes, CNRS, ISCR—UMR6226, F-35000 Rennes, France; abdeltif.amrane@univ-rennes.fr; 7 Centre de Recherche Scientifique et Technique en Analyses Physico-Chimiques (CRAPC), BP 384, Tipaza 42004, Algeria; medkebir@yahoo.fr; 8 Unité de Recherche en Analyses Physico-Chimiques des Milieux Fluides et Sols–(URAPC-MFS/CRAPC), 11, Chemin Doudou Mokhtar, Ben Aknoun 16100, Algeria; 9 Department of Biochemistry, College of Science, King Saud University, Riyadh 11451, Saudi Arabia; 10 School of Engineering, Merz Court, Newcastle University, Newcastle upon Tyne NE1 7RU, UK; jie.zhang@newcastle.ac.uk

**Keywords:** silver nanoparticles, green synthesis, biological activities, wastewater treatment

## Abstract

The green synthesis of silver nanoparticles (AgNPs) using plant extracts is an eco-friendly method with potential for biomedical and environmental applications. This study aims to synthesize silver nanoparticles (SO-AgNPs) using *Salvia officinalis* L. extract and evaluate their antioxidant and antibacterial properties, positioning them as candidates for applications in sustainable biomedicine and wastewater treatment. *S. officinalis* L. extract was used to synthesize AgNPs under optimized conditions, with a 10% extract/AgNO₃ ratio and a reaction time of 180 min. The SO-AgNPs were characterized using ATR-FTIR, XRD, SEM, DLS, and Zeta potential analysis. The antioxidant activity of the extract and SO-AgNPs was evaluated using ABTS^+•^ and DPPH^•^ radical scavenging assays. Antibacterial activity was tested against 11 bacterial strains and bacteria isolated from industrial effluent, with minimal inhibitory concentrations (MIC) determined for both the extract and SO-AgNPs. The SO-AgNPs demonstrated potent antioxidant activity, with IC₅₀ values of 0.233 mg/mL and 0.305 mg/mL in the ABTS^+•^ assay, and 0.173 mg/mL and 0.185 mg/mL in the DPPH^•^ assay for the extract and SO-AgNPs, respectively. Antibacterial testing showed MIC values of 0.25 mg/mL for SO-AgNPs and between 3.12 and 6.25 mg/mL for *S. officinalis* L. extract against *E. coli*, *P. aeruginosa*, *A. baumannii*, *MRSA*, *B. cereus*, and *S. epidermidis*. For bacteria isolated from industrial effluent, the MIC values were 0.125 mg/mL for SO-AgNPs and 0.5 mg/mL for the extract. This study highlights the dual antioxidant and antibacterial capabilities of *S. officinalis* L. extract and SO-AgNPs, demonstrating their potential for use in both biomedical and environmental applications, including wastewater treatment.

## 1. Introduction

In recent decades, there has been an increase in the incidence of microbial resistance against common antibiotics, and nanoparticles become more attractive as an alternative strategy to design and develop new antimicrobial drugs against multidrug-resistant strains [1]. Among various nanoparticles, silver nanoparticles (AgNPs) with particle sizes ranging from 1 to 100 nm have been widely used in biomedical applications such as drug delivery, medical imaging, wound dressing, and medicament for the promotion of wound healing [2]. AgNPs can be synthesized through various methods, yielding distinct morphologies and unique characteristics.

Traditional methods for synthesizing metallic nanoparticles often rely on complex and costly equipment and chemicals, raising environmental concerns. AgNPs can be produced using either ‘top-down’ or ‘bottom-up’ approaches, which may follow conventional or unconventional methods. The ‘top-down’ approach reduces bulk materials to nanoparticles via techniques such as pulse laser ablation, ball milling, pulse wire discharge, and evaporation-condensation, which vaporizes material into a carrier gas within a centrally placed furnace [3,4]. Alternatively, the ‘bottom-up’ approach assembles nanoparticles atom-by-atom, typically through chemical reduction. This method reduces Ag ions using agents like sodium citrate, Tollen’s reagent, ascorbate, etc., often with capping agents for size control. While efficient, it can involve toxic chemicals and produce non-eco-friendly byproducts [3,4].

Hence, there is a preference for “green” technologies in nanoparticle synthesis. These methods are simple, convenient, non-toxic, environmentally friendly, cost-effective, scalable, and offer greater stability than other approaches [5]. Green AgNPs synthesized using biological microorganisms [6], or plant extracts [7], are successfully employed as antimicrobial, antibiofilm, antifungal, anticancer, anti-angiogenic, attenuation renal damage therapy, anti-inflammatory, antioxidant, antileishmanial, and antiviral agents [8,9,10,11,12,13,14,15,16]. The extract of plants can be used as a reducing-stabilizing agent owing to the bioactive compounds such as phenols, organic acids, tannins, terpenoids, flavonoids, vitamins, and polysaccharides, which are capable of reducing silver ions to the elementary form (Ag^0^), where the properties of AgNPs strongly depend on their surface characteristics, such as morphology, size, and polydispersity [12].

The green synthesis of AgNPs using plant extracts has garnered significant attention due to its eco-friendly and sustainable approach. Among various plants, *Salvia officinalis* L., commonly known as sage, is recognized for its diverse range of bioactive compounds, including primary metabolites, such as polysaccharides and secondary metabolites like flavonoids and non-flavonoid compounds [17,18]. This rich chemical profile imparts sage with potent properties, making it effective in combating oxidative stress and bacterial infections. Moreover, the high content of bioactive substances in sage enhances its therapeutic potential and supports its role in the green synthesis of nanoparticles [17,18]. By leveraging these bioactive compounds, sage contributes to the development of AgNPs with improved stability and functionality, paving the way for advanced therapeutic applications and sustainable nanotechnology solutions.

Despite growing interest in green chemistry approaches for nanoparticle synthesis, there remains a significant knowledge gap concerning the optimal conditions for using *Salvia officinalis* L. extract in the green synthesis of AgNPs. Specifically, there is a lack of comprehensive studies examining the influence of synthesis parameters, such as time and extract/silver nitrate ratios on nanoparticle properties. Moreover, the dual functionality of these AgNPs—namely, their antioxidant activity against free radicals like DPPH^•^ and ABTS^+•^, and their antimicrobial efficacy against a wide spectrum of bacterial strains, including those isolated from industrial effluents—remains underexplored.

This research aims to address existing gaps by focusing on the green synthesis of AgNPs using *S. officinalis* L. extract (SO-AgNPs), with an emphasis on optimizing synthesis parameters to maximize their efficacy. The study systematically evaluates the antioxidant capacity of the synthesized nanoparticles through both DPPH^•^ and ABTS^+•^ assays, providing robust insights into their free radical scavenging potential. Additionally, their antibacterial activity is comprehensively assessed against 11 standard bacterial strains and isolates obtained from industrial wastewater effluent. By investigating both the antioxidant and antimicrobial properties of SO-AgNPs, this study not only enhances our understanding of the plant’s role in sustainable nanoparticle synthesis but also highlights its potential applications in biomedicine and eco-friendly wastewater treatment. Ultimately, this work contributes to advancing environmental sustainability and fostering progress in biomedical research.

## 2. Results

### 2.1. Biosynthesis of AgNPs Using S. officinalis L. Leaves Extract

**Figure 1:**
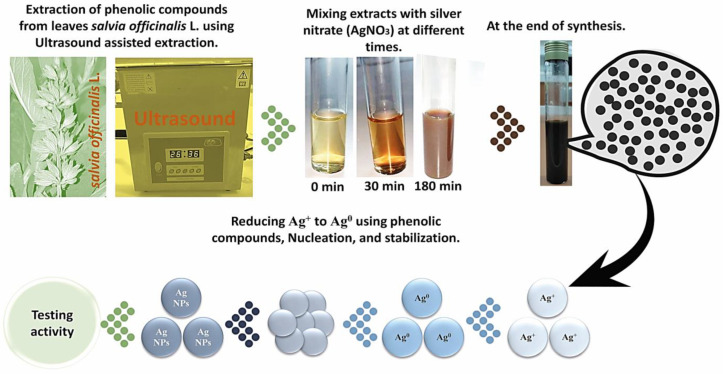
Biosynthesis of SO-AgNPs over time using *S. officinalis* L. extract.

**Figure 2:**
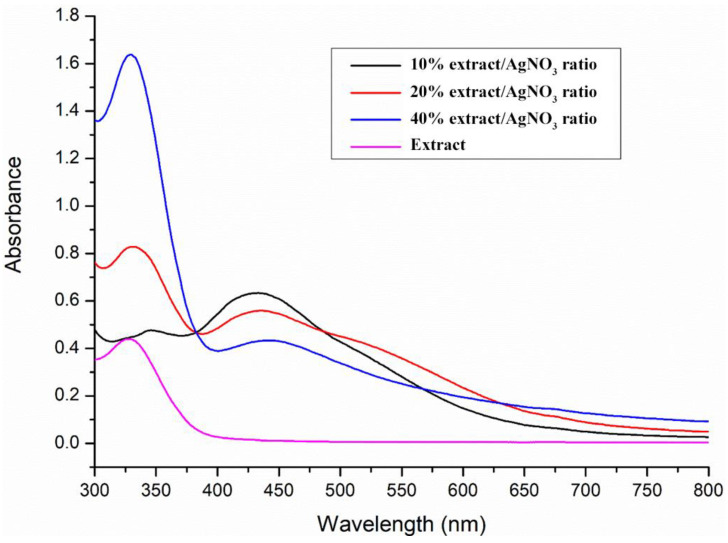
Extract/Silver nitrate ratio effect on the biosynthesis of SO-AgNPs at 180 min.

The optimal extract of *S. officinalis* L. leaves was employed for the green synthesis of SO-AgNPs (Figure 1). Figure 2 shows the effect of the extract/AgNO_3_ ratio on the biosynthesis of SO-AgNPs, with 10%, 20%, and 40% ratios studied over a defined time of 180 min. The synthesis reaction was incubated in the absence of light and the color changes in the reaction mixture from pale yellow to brownish indicated the formation of SO-AgNPs, through the reduction in Ag^+^ ions to Ag^0^ nanoparticles. In addition, the UV-visible spectra analysis of SO-AgNPs displayed two distinct peaks at 425 nm and 325 nm, corresponding to SO-AgNPs and *S. officinalis* extract, respectively.

Figure 2 demonstrates that the extract/AgNO_3_ ratio significantly affects SO-AgNPs synthesis. The maximum intensity of the peak at 425 nm was obtained for the 10% extract/AgNO_3_ ratio, followed by the 20% and 40% ratios, respectively. This suggested that a lower concentration of the extract facilitates more efficient nanoparticle formation, likely due to the optimal availability of reducing agents at lower ratios. On the other hand, the peak at 325 nm, was observed for both 20% and 40% ratios at 180 min of synthesis time, indicating the presence of unreacted bioactive compounds, revealing incomplete consumption of the extract for nanoparticle synthesis at higher concentrations.

**Figure 3:**
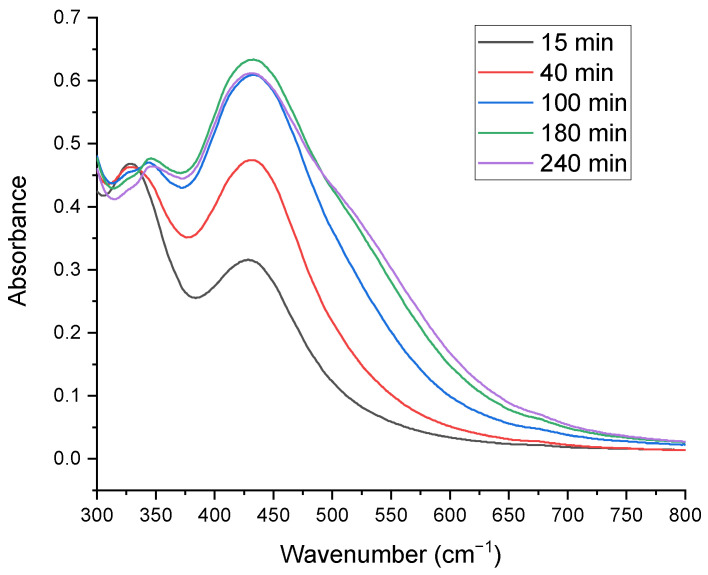
Time effect on the biosynthesis of SO-AgNPs using a 10% extract/AgNO_3_ ratio.

Additionally, Figure 3 evaluates the impact of synthesis time (15, 40, 100, 180, and 240 min) on SO-AgNPs formation using the UV-Vis spectral analysis technique. The formation of SO-AgNPs increased remarkably with synthesis time increasing from 15 min to 180 min, as evidenced by the increasing peak intensity at 425 nm. Beyond 180 min, there was no significant change in the intensity of the SO-AgNPs peak, indicating the completion of the reaction at this time point.

Overall, these results highlight the importance of optimizing both the extract/AgNO_3_ ratio and the synthesis time to achieve efficient and complete biosynthesis of SO-AgNPs using *S. officinalis* L. extract. 

### 2.2. Characterization of Green Synthesized SO-AgNPs

**Figure 4:**
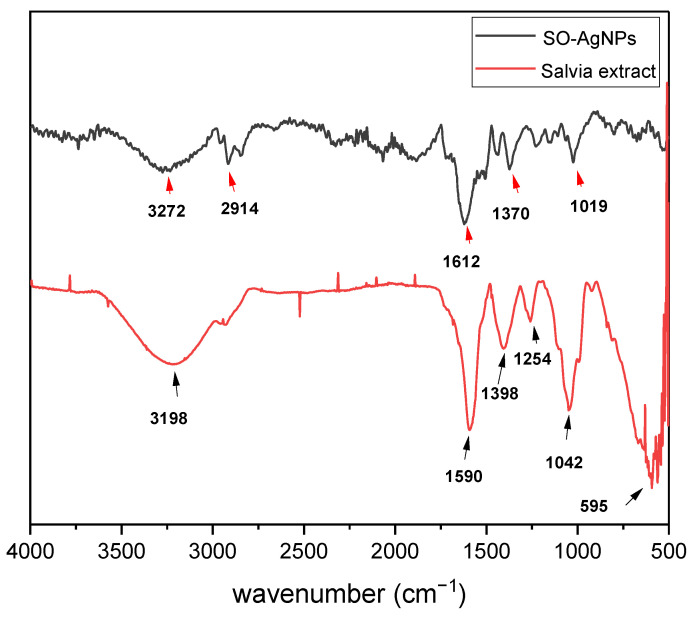
ATR-FTIR spectrum of *Salvia* extract and SO-AgNPs obtained under optimal conditions of synthesis.

The ATR-FTIR spectrum of *S. officinalis* L. extract (Figure 4) reveals distinct functional groups that play a crucial role in the green synthesis of SO-AgNPs. A broad peak at 3198 cm⁻¹ corresponds to O-H stretching vibrations, indicating the presence of hydroxyl groups in phenolic compounds. The peak at 1590 cm⁻¹ is associated with C=C stretching in aromatic rings, highlighting the abundance of aromatic compounds in the plant extract. Additionally, peaks at 1254 and 1042 cm⁻¹ are attributed to C-O stretching vibrations in alcohols or esters.

The ATR-FTIR spectrum of SO-AgNPs synthesized using *S. officinalis* (Figure 4) demonstrates significant differences compared to the plant extract, reflecting the successful reduction and stabilization of silver nanoparticles by bioactive compounds from the extract. Notable peaks include the one at 2914 cm⁻¹, indicative of C-H stretching vibrations of the aliphatic hydrocarbons, suggesting the presence of organic components from the synthesis process (Figure 4) [19]. A peak at 1612 cm⁻¹, associated with the C=C stretching vibrations in aromatic compounds, indicates the presence of aromatic groups, likely originating from the plant extract or added reagents [20]. Another peak observed at 1370 cm⁻¹, corresponding to C-H bending vibrations in methyl groups, points to methyl-containing functional groups in the nanoparticles themselves [21]. Additionally, a peak at 1019 cm⁻¹ can be related to C-O stretching vibrations in alcohols or ethers, suggesting the presence of oxygen-containing functional groups in the nanoparticles [19]. Notably, a peak at 411 cm⁻¹ is attributed to Ag-O stretching vibrations, indicating the presence of silver oxide or silver-containing compounds with oxygen-containing functional groups (Figure 4).

The comparison between the two spectra highlights the involvement of bioactive compounds in *S. officinalis* extract in reducing and capping the nanoparticles. The shift and disappearance of certain peaks in the SO-AgNPs spectrum, such as the attenuation of the O-H band at 3198 cm⁻¹ and the emergence of Ag-O stretching at 411 cm⁻¹, provide strong evidence of the interaction between plant-derived molecules and silver ions, resulting in the successful synthesis of silver nanoparticles. This analysis underscores the dual role of *S. officinalis* extract as both a reducing agent and a stabilizer in the green synthesis process.

**Figure 5:**
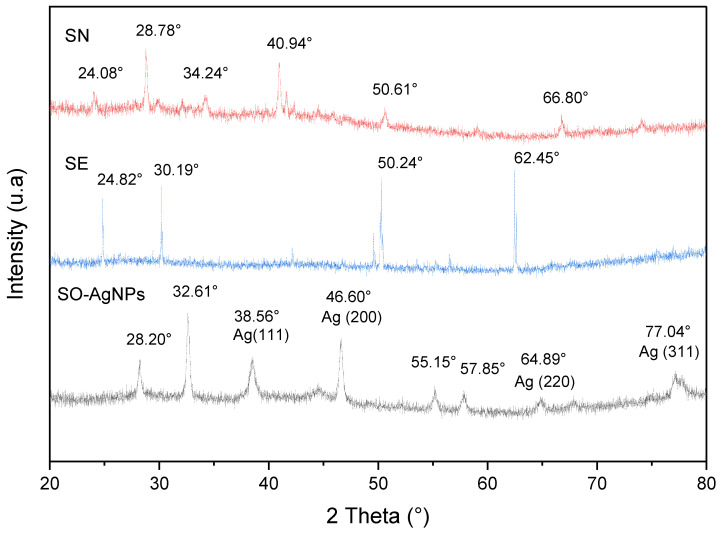
XRD pattern of SO-AgNPs, SN (Silver nitrate), and SE (*Salvia* extract) obtained under optimal conditions of synthesis.

The XRD arrays presented in Figure 4 aim to compare the diffraction patterns of silver nitrate (SN), *S. officinalis* L. extract (SE), and SO-AgNPs synthesized using *S. officinalis* L. extract (SO-AgNPs). The pattern for silver nitrate reveals sharp, intense peaks at 28.78°, 34.24°, 40.94°, 50.61°, and 66.80°, corresponding to the (111), (200), (220), (311), and (222) planes of face-centered cubic (FCC) silver, respectively, confirming the presence of crystalline silver nitrate with an FCC structure (Figure 5). In contrast, the *S. officinalis* L. extract exhibits broad, diffuse peaks, indicating its amorphous nature, which is typical for plant extracts that contain a complex mixture of organic compounds. The XRD pattern for the SO-AgNPs shows sharp, intense peaks at 28.20°, 38.56°, 46.60°, 55.15°, 57.85°, 64.89°, and 77.04°, corresponding to the (111), (200), (220), (311), (222), (331), and (400) planes of FCC silver, respectively, according to the JCPDS, No. 4-0783 (Figure 5), where our results are in accordance with the findings in [22,23].

This pattern suggests the successful formation of crystalline AgNPs with a similar face-centered cubic structure to the silver nitrate precursor (Figure 5). However, the presence of additional peaks in the SO-AgNPs pattern compared to SN implies that the nanoparticles might differ slightly in crystallite size or strain. Notably, the absence of peaks related to the organic components of the plant extract in the SO-AgNPs pattern indicates that the synthesis process effectively removed or converted these components during nanoparticle formation (Figure 5).

**Figure 6:**
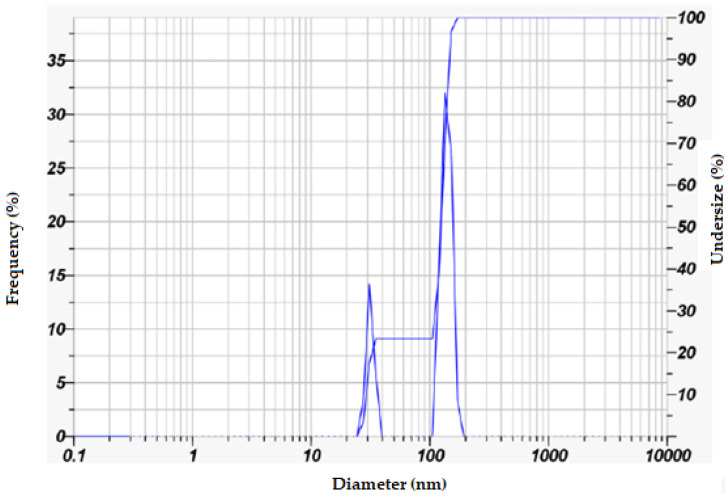
Zeta potential analysis of SO-AgNPs.

To examine the size of SO-AgNPs synthesized using *Salvia* extract, a dynamic light scattering (DLS) analysis using Horiba Scientific SZ-100 Nanopartica line equipment (Horiba Ltd., Kyoto, Japan), based on the concept of Brownian motion of particles, was carried out to analyze the intensity of scattered light as it interacts with the suspended particles in a water medium to determine the particle size distribution. The analysis data reveals a polydisperse nature, characterized by two distinct size populations. The primary size distributions include nanoparticles with a mean size of 29.6 nm and a mode of 29.5 nm and a second, larger group with a mean size of 130.5 nm and a mode of 129.0 nm. The overall mean particle size is 107.1 nm, with a Z-average of 111.7 nm, and a polydispersity index (PI) of 0.320, indicating a moderately broad size distribution (Table 1 and Figure 6). The zeta potential measurement of −21.2 mV indicates that the nanoparticles possess a negative surface charge, which confers moderate stability to the suspension, although there is some potential for aggregation. The electrophoretic mobility value of −0.000164 cm^2^/Vs aligns with the zeta potential, confirming the negative charge on the nanoparticles (Table 1 and Figure 6). These findings suggest that the synthesis method using *Salvia* extract effectively produces SO-AgNPs with a significant range of sizes, offering a balance between stability and potential aggregation, suitable for various applications that benefit from these specific nanoparticle characteristics.

**Figure 7:**
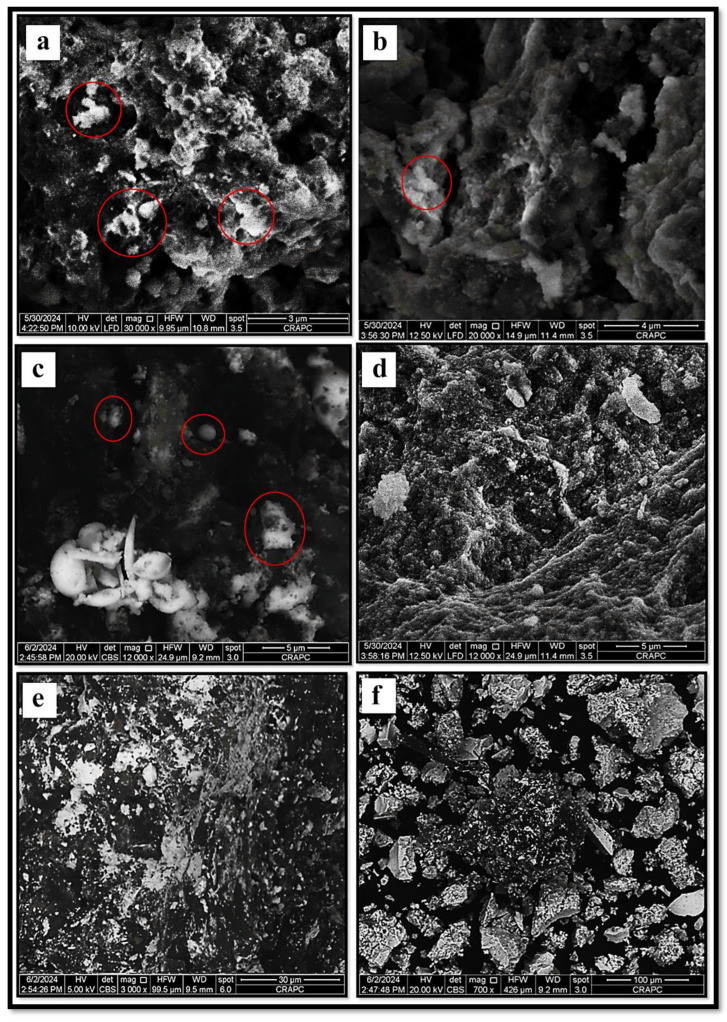
SEM microscopy micrographs of SO-AgNPs captured under varied imaging magnifications, (**a**) ×30,000, HFW: 9.95 μm, WD: 10.8 mm, (**b**) ×20,000, HFW: 14.9 μm, WD: 11.4 mm, (**c**) ×12,000, HFW: 24.9 μm, WD: 9.2 mm, (**d**) ×12,000, HFW: 24.9 μm, WD: 11.4 mm, (**e**) ×3000, HFW: 99.5 μm, WD: 9.5 mm, (**f**) ×700, HFW: 426.0 μm, WD: 9.2 mm. The red circles highlighting the SO-AgNPs at each magnification level.

Furthermore, the SEM images (Figure 7) confirm the successful synthesis of SO-AgNPs. The micrographs reveal distinct, well-defined nanoparticle structures with varying sizes and shapes, along with evidence of aggregation, characteristic of AgNPs. The clear visibility of these particles under different magnifications and imaging conditions indicates the formation of stable, morphologically consistent nanoparticles, validating the effectiveness of the synthesis process.

### 2.3. Scavenging Free Radical Activity of Slavia Extract and SO-AgNPs

The antioxidant activity of various substances is critical in combating oxidative stress, which can lead to numerous chronic diseases. Both natural extracts and engineered nanoparticles have been studied for their potential to neutralize free radicals. Salvia extract is known for its rich composition of bioactive compounds with strong antioxidant properties [18]. Similarly, SO-AgNPs have gained attention for their unique physicochemical properties and potential health benefits, including antioxidant effects. This study aims to compare the antioxidant activities of *Salvia* extract and SO-AgNPs using DPPH^•^ and ABTS^+•^ free radical scavenging assays. 

**Figure 8:**
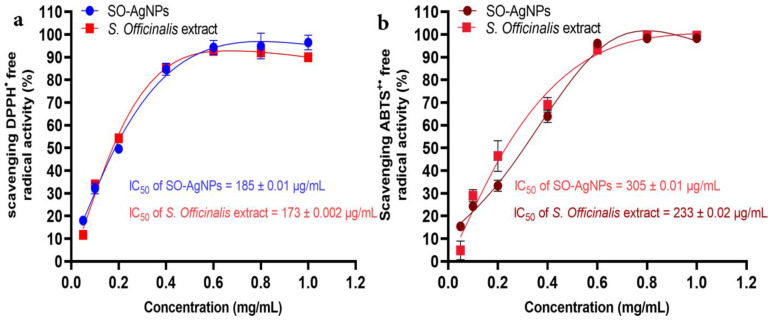
Antioxidant activity of both *S. officinalis* L. extract and SO-AgNPs against DPPH^•^ (**a**) and ABTS^+•^ (**b**) free radical.

Figure 8 illustrates that both *Salvia* extract and SO-AgNPs possess significant antioxidant activities against DPPH^•^ and ABTS^+•^ free radicals, although with some differences in their efficacy. While Salvia extract performs slightly superior, SO-AgNPs exhibit notable antioxidant properties. For the DPPH^•^ assay (Figure 8a), SO-AgNPs achieve a substantial free radical scavenging activity, with an IC_50_ value of 0.185 ± 0.01 mg/mL, which is quite close to that of *Salvia* extract (0.173 ± 0.002 mg/mL).

This indicates that SO-AgNPs are highly effective at neutralizing DPPH^•^ radicals, achieving over 80% scavenging activity at higher concentrations. Similarly, in the ABTS^+•^ assay (Figure 8b), SO-AgNPs demonstrate a significant antioxidant effect with an IC_50_ value of 0.305 ± 0.01 mg/mL. Despite the Salvia extract showing a lower IC_50_ (0.233 ± 0.02 mg/mL), SO-AgNPs still display a strong capability to scavenge ABTS^•+^ radicals, particularly at higher concentrations, reaching nearly 90% activity. These findings confirm that SO-AgNPs, while slightly less potent than *salvia* extract, are nonetheless effective antioxidants and represent a viable option for free radical scavenging, potentially contributing to developing novel antioxidant therapies.

### 2.4. Antimicrobial Activity of SO-AgNPs

**Figure 9:**
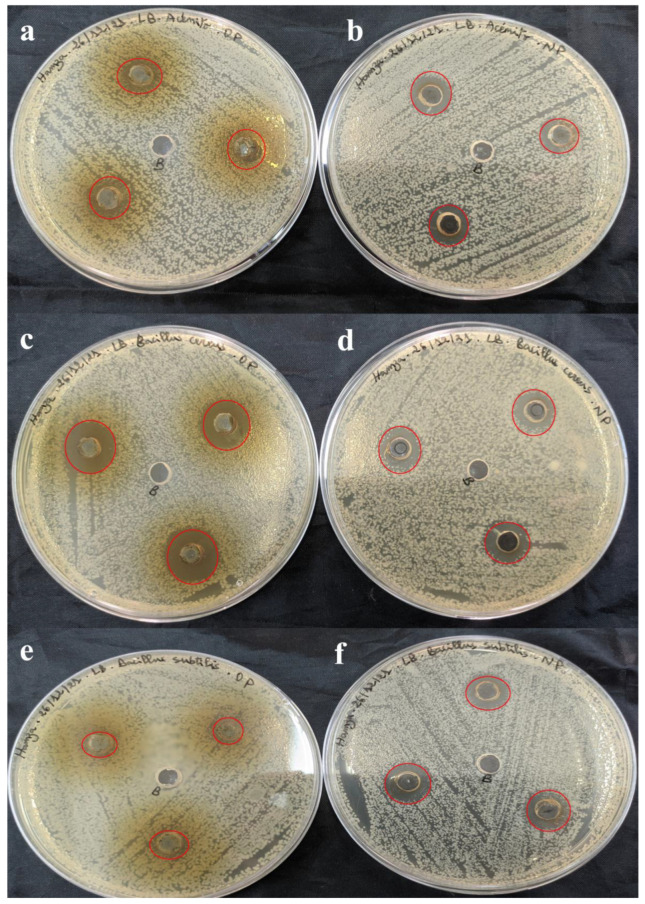
Antimicrobial activity of *S. officinalis* L. extract (**a**,**c**,**e**) and SO-AgNPs (**b**,**d**,**f**) against *A. baumannii*, *B. cereus*, and *B. subtilis*, respectively. The red circles indicate the zones of inhibition surrounding the tested samples and the negative control.

**Figure 10:**
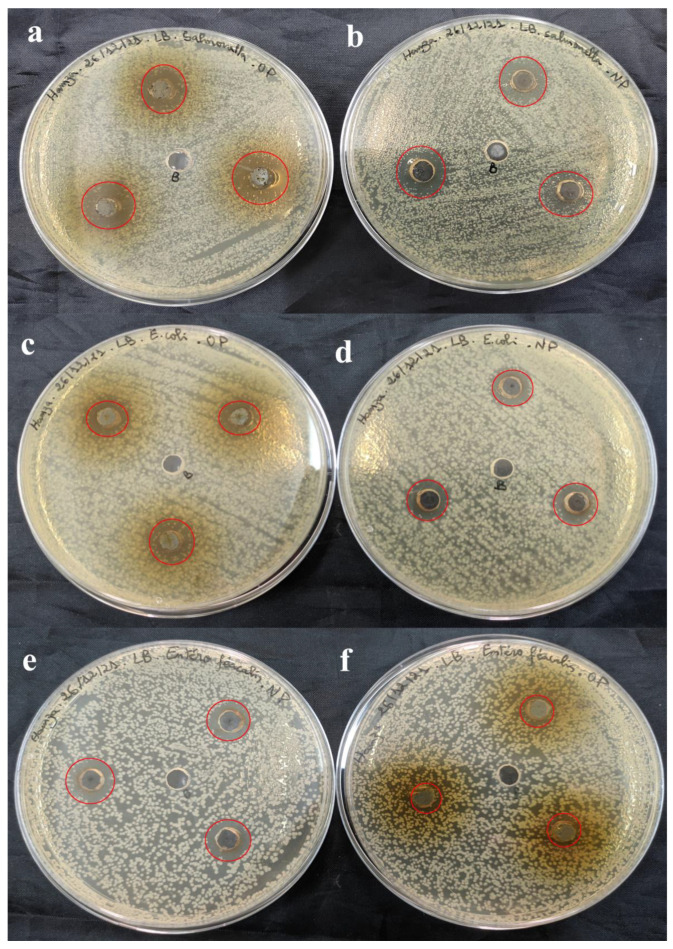
Antimicrobial activity of *S. officinalis* L. extract (**a**,**c**,**f**) and SO-AgNPs (**b**,**d**,**e**) against *Salmonella* spp., *E. coli*, and *E. faecalis*, respectively. The red circles indicate the zones of inhibition surrounding the tested samples and the negative control.

**Figure 11:**
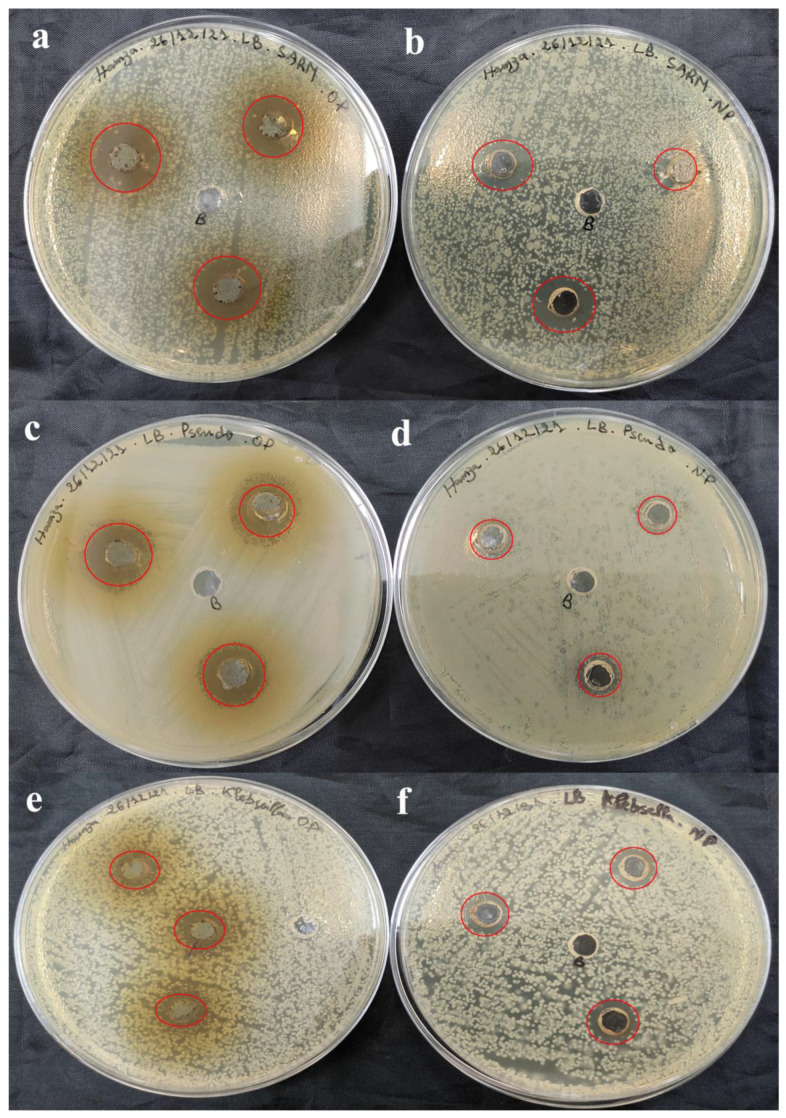
Antimicrobial activity of *S. officinalis* L. extract (**a**,**c**,**e**) and SO-AgNPs (**b**,**d**,**f**) against *MRSA*, *P. aeruginosa*, and *K. pneumoniae*, respectively. The red circles indicate the zones of inhibition surrounding the tested samples and the negative control.

**Figure 12:**
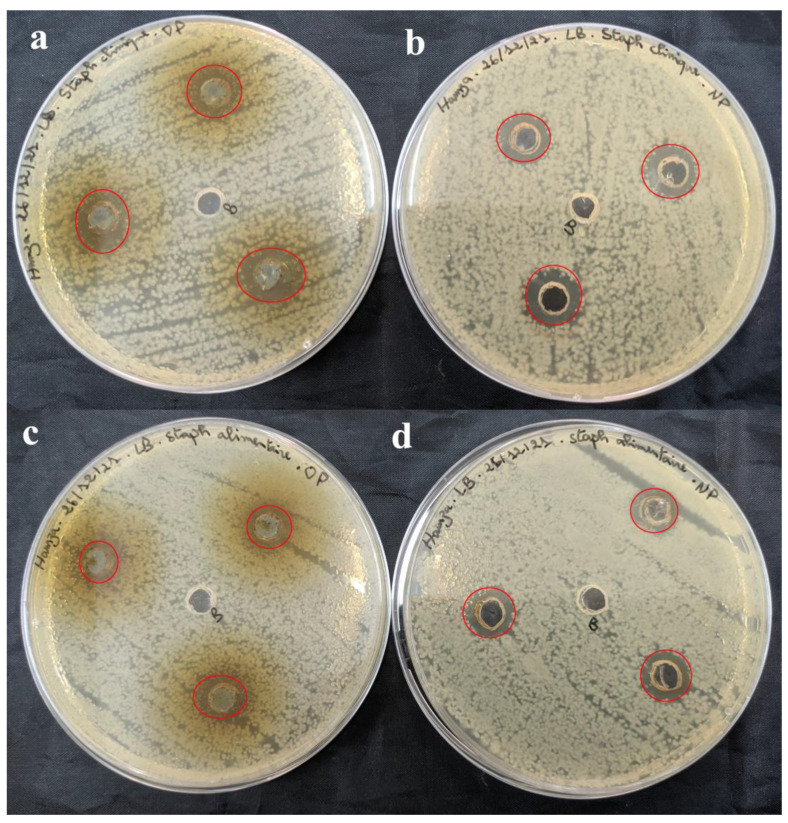
Antimicrobial activity of *S. officinalis* L. extract (**a**,**c**) and SO-AgNPs (**b**,**d**) against *S. epidermidis*, and *S. aureus*, respectively. The red circles indicate the zones of inhibition surrounding the tested samples and the negative control.

The antimicrobial activity of SO-AgNPs and *S. officinalis* L. leaves extract at different concentrations was investigated against eleven pathogenic bacteria strains (both Gram-positive and Gram-negative) using the agar well diffusion assay (Table 2 and Figure 9, Figure 10, Figure 11 and Figure 12). The zones of inhibition (mm) around each well were measured and illustrated in detail in Table 2 and Figure 9, Figure 10, Figure 11 and Figure 12.

Table 2 shows that the extract prepared at 50 mg/mL was effective against Gram-positive pathogenic strains including *B. cereus* (18.33 ± 1.15 mm) (Figure 9c), *MRSA* (16.67 ± 1.52 mm) (Figure 11a), and *E. faecalis* (11.33 ± 0.57 mm) (Figure 10e). Notably, the extract did not affect the pathogenic *B. subtilis (*Figure 9e), *S. aureus (*Figure 12c), and *S. epidermidis* (Figure 12a) as no inhibition zones were observed. This indicates that the extract’s antimicrobial components were ineffective against *B. subtilis*, *S. aureus*, and *S. epidermidis* possibly due to its unique resistance mechanisms or the specific composition of its cell wall.

Furthermore, the extract at 50 mg/mL also exhibited antimicrobial activity against 2 Gram-negative pathogenic strains, including *P. aeruginosa* (15 mm) (Figure 11c), and *Salmonella* spp. (15.67 ± 2.88 mm) (Figure 10a). These results suggest that the *S. officinalis* L. extract contains compounds capable of permeating the outer membrane of Gram-negative bacteria and disrupting their cellular processes (Table 2 and Figure 9, Figure 10, Figure 11 and Figure 12). However, the extract demonstrated no inhibitory effects against *A. baumannii* (Figure 10a), *E. coli* (Figure 10c), and *K. pneumoniae* (Figure 11e), indicating a selective antimicrobial spectrum.

Comparatively, SO-AgNPs synthesized using the extract of *S. officinalis* L. demonstrated superior antimicrobial activity to the extract alone. At 1 mg/mL concentration, SO-AgNPs showed positive activity against all tested Gram-positive and Gram-negative pathogenic strains. The inhibition zones for SO-AgNPs ranged from 11.67 mm to 15 mm. For the six Gram-positive bacteria strains, the inhibition zones were *B. cereus* (14.67 ± 0.57 mm) (Figure 9d), *B. subtilis* (15 mm) (Figure 9f), *MRSA* (14.67 ± 1.52 mm) (Figure 11b), *S. epidermidis* (14.67 ± 0.57 mm) (Figure 12b), and *S. aureus* (14.67 ± 0.57 mm) (Figure 12d). However, SO-AgNps showed no antimicrobial activity against *E. faecalis* (Figure 10f). 

Additionally, SO-AgNPs at 1 mg/mL also showed antimicrobial activity against the five Gram-negative pathogenic strains, including *E. coli* (15 mm) (Figure 10d), *A. baumannii* (15 mm) (Figure 9b), *K. pneumoniae* (14 ± 1 mm) (Figure 11f), and *Salmonella* spp. (15 ± 1 mm) (Figure 10b). Furthermore, SO-AgNPs exhibited no activity against *P. aeruginosa* (Figure 11d). These results were in agreement with the results of [14], which found that SO-AgNPs exhibited positive antimicrobial activity against *S. aureus* and *E. coli.*

**Figure 13:**
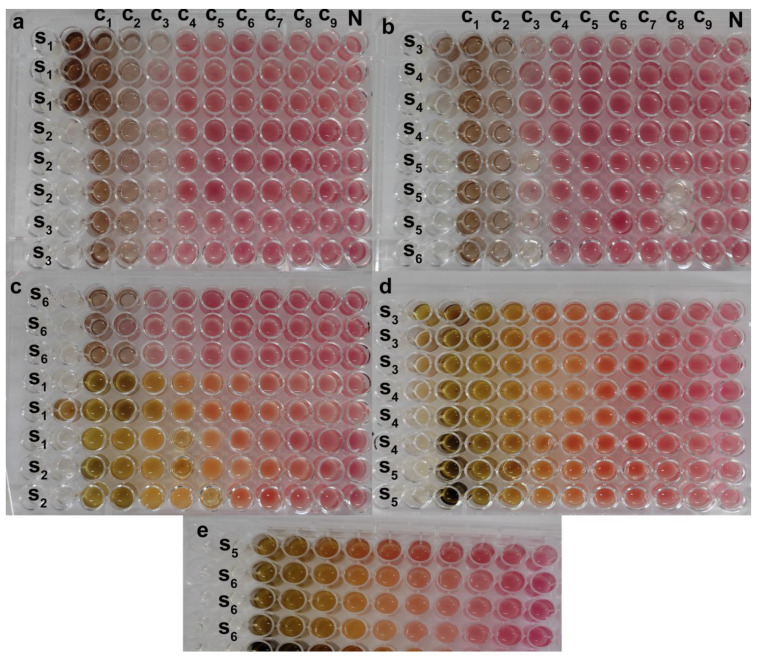
The MIC of SO-AgNps (**a**–**c** (S_6_)) and *Salvia* extract (c S_1_-S_2_, **d**,**e**) using the tetrazolium red salt (TTC) colorimetric assay, C_1_–C_9_ are the concentration of *salvia* extract (25–0.976 mg/mL) and SO-AgNps (0.5–0.001 mg/mL), N is the negative control, S_1_ is *E. coli*, S_2_ is *P. aeruginosa*, S_3_ is *A. baumannii*, S_4_ is *MRSA*, S_5_ is *B. cereus*, S_6_ is *S. epidermidis*.

The MIC results demonstrate that SO-AgNPs exhibit significantly stronger antimicrobial effects compared to the *S. officinalis* L. extract alone against various pathogenic bacteria. SO-AgNPs show a consistent MIC of 0.25 mg/mL against *E. coli*, *P. aeruginosa*, *A. baumannii*, *MRSA*, *B. cereus*, and *S. epidermidis* (Table 2, Figure 13). This low MIC value indicates a potent antimicrobial activity, likely due to the synergistic effect between silver ions and the bioactive compounds of *S. officinalis* L., which together enhance the ability to disrupt bacterial cell walls, interfere with microbial enzyme functions, and inhibit bacterial growth.

In contrast, the *S. officinalis* L. extract alone requires significantly higher concentrations (ranging from 3.12 to 6.25 mg/mL) to achieve similar inhibitory effects, indicating a comparatively lower antimicrobial potency. For instance, *S. epidermidis* and *E. coli* have MIC values of 3.12 mg/mL and 6.25 mg/mL with the plant extract alone, respectively, suggesting that while the extract has inherent antimicrobial properties, it is less effective than when combined with silver nanoparticles (Table 2, Figure 13). This enhanced efficacy of SO-AgNPs highlights their potential as a robust antimicrobial agent, particularly valuable in targeting multidrug-resistant bacterial strains, thus presenting a promising strategy for developing alternative treatments in antimicrobial therapies (Table 2, Figure 13).

Furthermore, an antimicrobial assessment of SO-AgNPs, silver nitrate, and *S. officinalis* L. extract against microbial contaminants isolated from dairy industry effluents was also conducted. The results demonstrated significant variations in antibacterial activity among the three agents, with inhibition zones ranging from 2 mm to 9 mm, indicating their differing efficacy in targeting effluent-associated pathogens. Notably, the SO-AgNPs nanoparticles exhibited the strongest antibacterial effects, reflected in a minimal inhibitory concentration (MIC) of 0.125 mg/mL, compared to 0.5 mg/mL for the *S. officinalis* L extract. These findings emphasize the superior potency of biosynthesis SO-AgNPs in reducing microbial contamination in industrial effluents, indicating their potential for effective implementation in the protection of environmental systems and wastewater treatment processes.

## 3. Discussion

Green synthesis of nanoparticles, utilizing *S. officinalis* L. extract as reducing and stabilizing agents, offers an eco-friendly and cost-effective alternative to conventional chemical methods. In our study, the peak at 425 nm in the UV-visible spectra indicates the successful formation of SO-AgNPs through the surface plasmon resonance (SPR) of electrons on the nanoparticles’ surface, as shown in Figure 1, Figure 2 and Figure 3 [24,25]. This result aligns with previous research by Aboelfetoh et al. [26], who observed that using extract concentrations below 20% in a 1 mM AgNO_3_ solution enhances SPR intensity and shifts the SPR peak to a lower wavelength (435 nm), reflecting a reduction in the mean size of the nanoparticles. However, their study also noted that concentrations exceeding 25% result in diminished SPR intensity due to nanoparticle aggregation, which reduces the nanoparticles’ functional properties and their economic value [27]. 

The variations in peak intensities and positions reveal the intricate relationship between extract concentration, silver nitrate concentration, and reaction time. Lower extract concentrations (10%) facilitate a more effective synthesis of SO-AgNPs, likely due to improved stabilization and reduction efficiency. Conversely, higher extract concentrations (20% and 40%) may lead to nanoparticle aggregation or incomplete reduction, as evidenced by the additional peak at 325 nm. On the other hand, the optimal synthesis time for maximum nanoparticle yield is 180 min, beyond which no further enhancement in nanoparticle formation occurs. These findings are consistent with the results reported in [10]. Optimizing both the extract/AgNO_3_ ratio and synthesis time is crucial for achieving efficient and complete biosynthesis of SO-AgNPs.

The comprehensive characterization of SO-AgNPs was carried out using ATR-FTIR, XRD, and particle size and zeta potential analyses. The ATR-FTIR spectrum revealed the presence of various functional groups, including aliphatic hydrocarbons, aromatic compounds, and oxygen-containing groups, indicating the involvement of organic components and silver oxide. XRD analysis confirmed the crystalline nature of the SO-AgNPs with a face-centered cubic (FCC) structure similar to the silver nitrate precursor, while also showing additional peaks suggesting differences in crystallite size or strain. Particle size distribution analysis identified a polydisperse nature of the nanoparticles with a mean size of 107.1 nm and a moderate polydispersity index, reflecting variability in particle size and this value corresponds to the particle agglomeration. The zeta potential measurement indicated a negative surface charge of −21.2 mV, suggesting stability of the nanoparticle suspension with some potential for aggregation.

The enhanced antimicrobial efficacy of SO-AgNPs can be attributed to their dual mechanism of action, which combines physical and chemical disruption of bacterial cells. The agar well diffusion assay results show that SO-AgNPs exhibit significantly superior antimicrobial activity compared to the plant extract alone. At a concentration of 1 mg/mL, SO-AgNPs effectively inhibited the growth of all tested Gram-positive and Gram-negative bacterial strains, with inhibition zones ranging from 11.67 mm to 15 mm, except for *P. aeruginosa* and *K. pneumoniae*, which showed no activity. This comprehensive activity contrasts with the extract alone, which, at 50 mg/mL, was effective against *B. cereus*, *MRSA*, *P. aeruginosa*, *E. faecalis*, and *Salmonella* spp. In comparison to previous studies on AgNPs synthesized using various plant extracts, SO-AgNPs demonstrate a promising antimicrobial profile, with inhibition zones ranging from 11.67 mm to 15 mm across different bacterial strains (Table 2). Notably, these values are higher than those reported for *Symphyti radix* AgNPs, which showed inhibition zones between 3.10 mm and 5.40 mm, indicating that SO-AgNPs possess stronger antimicrobial activity (Table 3).

Similarly, SO-AgNPs exhibit comparable or slightly higher inhibition zones than *Calendula officinalis* AgNPs, whose zones ranged from 10.50 mm to 15.10 mm. They are generally on par with *Hyssopus officinalis* AgNPs, which showed inhibition from 9.50 mm to 16.50 mm. Compared to *S. officinalis* AgNPs from [29], which reported zones between 9.5 mm and 17.75 mm, SO-AgNPs demonstrate similar efficacy, particularly against *E. coli* and *P. aeruginosa*, where they achieve inhibition zones of 14 mm or higher, exceeding prior results (Table 3). However, studies on *Eucalyptus globulus* and *S. officinalis* AgNPs in [30] showed larger inhibition zones, ranging from 18.8 mm to 24.4 mm, suggesting that the synthesis methods, nanoparticle size, and unique phytochemical composition of each plant extract significantly influence antimicrobial potency (Table 3). Thus, while SO-AgNPs display substantial antimicrobial activity, certain plant-derived AgNPs with alternative synthesis protocols may yield even greater efficacy. This underscores the importance of synthesis optimization and phytochemical selection in enhancing AgNPs antimicrobial properties.

The observed antimicrobial enhancement in SO-AgNPs is largely due to the electrostatic attraction between the negatively charged bacterial cell membranes and the positively charged nanoparticles. This interaction facilitates nanoparticle adherence to bacterial cells, causing structural damage and increased membrane permeability. Concurrently, the release of silver ions from the SO-AgNPs further disrupts bacterial cellular processes by interacting with critical biomolecules, such as proteins, lipids, and DNA, leading to bacterial dysfunction and death [9]. The smaller size and larger surface area of the SO-AgNPs enhanced their interaction with bacterial cells, improving the efficacy of these mechanisms. The significantly lower MIC of 0.25 mg/mL for SO-AgNPs compared to 3.12 to 6.25 mg/mL for the *S. officinalis* extract against *E. coli*, *P. aeruginosa*, *A. baumannii*, *MRSA*, *B. cereus*, and *S. epidermidis* underscores the nanoparticles’ increased potency (Figure 13) [33,34]. These results demonstrate that the combination of physical disruption by nanoparticles and chemical disruption by silver ions results in a broader spectrum of activity and more effective antimicrobial action.

Moreover, the superior potency of SO-AgNPs, reflected in their lower MIC value of 0.125 mg/mL against dairy effluent-associated pathogens compared to 0.5 mg/mL for the plant extract, can be attributed to several key factors. The antibacterial efficacy of SO-AgNPs is known to depend on their size, shape, synthesis method, and the composition of the capping agents that stabilize the nanoparticles [35]. The bioactive compounds in the *Salvia* extract likely serve as effective capping agents, enhancing the stability and bioactivity of the nanoparticles. Additionally, the small size of SO-AgNPs provides a larger surface area, increasing their interaction with bacterial cells and facilitating their antimicrobial action. These results demonstrate the significant potential of biogenic SO-AgNPs synthesized from *S. officinalis* in mitigating microbial contamination, particularly in industrial effluent treatment processes where enhanced antimicrobial stability and efficacy are crucial.

## 4. Materials and Methods

### 4.1. Materials

Ethanol and silver nitrate (AgNO_3_) were purchased from SIGMA-ALDRICH (St. Louis, MO, USA). Nutrient Agar, Luria Agar (LA) (tryptone, yeast Extract, and sodium chloride (NaCl)) were obtained from Liofilchem (Roseto degli Abruzzi, Teramo, Italy). All reagents were utilized without any modification.

### 4.2. Ultrasound-Assisted Extraction

Fresh leaves of *S. officinalis* L. were harvested in April 2021 from Ain Bessem, Bouira province, Algeria (latitude 36.325295, longitude 3.674675, altitude 690 m). Phenolic compounds were extracted using an ultrasound cleaning bath (J.P. SELECTA, s.a, Barcelona, Spain, SN. 3000865) operating at 40 kHz, with a power generator of 120 W and a power heater of 75 W. The bath had cavity dimensions of 15 × 24 × 14 cm (H/W/D). The extraction followed the optimal conditions [17]. Specifically, 1 g of *S. officinalis* L. powder (particle size 200 µm) was mixed with 30 mL of a 52% ethanol-water mixture and sonicated for 10 min at 60 °C. The extract was then filtered using centrifugation (Sigma 3–16L, Osterode, Germany) at 5000 rpm for 10 min to remove insoluble materials and equilibrate to the final volume. The resulting *S. officinalis* L. extract was lyophilized and stored at 4 °C for further analysis.

### 4.3. Green Synthesis of SO-AgNPs

For the biosynthesis of SO-AgNPs, an aqueous extract of *S. officinalis* L. was prepared by dissolving 5 mg/mL of the lyophilized powder of the optimal extract in deionized water. This prepared extract was then added to a 1 mM solution of AgNO_3_, with extract/silver nitrate ratios of 10%, 20%, and 40% (*v*/*v*) and synthesis times of 15, 40, 100, 180, and 240 min being studied, as shown in Figure 1, Figure 2 and Figure 3. The biosynthesis was conducted in a shaking water bath at 60 °C and 80 rpm (Maxturdy-18, 10003001653005, DAIHAN Scientific Co., Ltd., Wonju, Gangwon Province, Republic of Korea). The initial detection of the synthesized SO-AgNPs was indicated by a color change from pale yellow to dark brown. The reduction in Ag^+^ to Ag^0^ nanoparticles was monitored using extract/nitrate mixtures (1/10 *v*/*v*) at wavelengths of 300 to 800 nm with a UV-visible spectrophotometer, as depicted in Figure 2 and Figure 3. From these figures, the optimal extract/silver nitrate ratio was found to be 10%, with a synthesis time of 180 min. These optimal parameters were then explored for further study. The SO-AgNPs formed were obtained by centrifugation at 9000× *g* for 10 min at 25 °C. The precipitated product was washed several times with deionized water, and the SO-AgNPs were dispersed in deionized water and lyophilized for further experiments.

### 4.4. Characterization of SO-AgNPs

The functional groups and chemical interactions in SO-AgNPs were characterized using the method outlined in Kebir et al. [36]. This analysis was conducted with a Bruker Alpha 1 infrared spectrometer equipped with Diamond crystal an attenuated total reflectance (ATR) cell. The infrared spectrum was recorded over a wavenumber range of 4000 to 400 cm⁻¹.

The crystalline structures of *Salvia* extract, silver nitrate, and SO-AgNPs were analyzed using a Bruker D2 X-ray diffractometer (Bruker Corp., Billerica, MA, USA) [37]. This instrument employed Cu-Kα radiation with a wavelength of 1.5404 Å, operating at 45 kV and 40 mA. Measurements were taken with a step size of 0.0262° over a diffraction angular range of 5° to 75° in continuous scan mode. Crystallographic parameters of the samples were determined using X-Pert HighScore software (version 3.0e (3.0.5)), and the crystallite sizes were calculated based on the Scherrer equation.

The size of SO-AgNPs was determined using the Horiba SZ-100 DLS instrument (Horiba Ltd., Kyoto, Japan) [36]. Samples dispersed in 0.895 mPa·s viscosity medium were measured at 90° scattering angle and 25.0 °C, with transmission intensity and count rate recorded. The Horiba SZ-100 software (version 2. 20) was used for analyzing data to establish particle size distribution. For the zeta potential of SO-AgNPs, samples in 0.896 mPa·s viscosity and 0.189 mS/cm conductivity medium were tested at 25.0 °C with 3.4 V electrode voltage. The instrument assessed electrophoretic mobility and zeta potential, with the software used to evaluate stability and surface charge.

The SEM analysis of SO-AgNPs was performed in normal secondary electron (SE) mode under low-vacuum conditions. Various imaging parameters, including large field detector (LFD) settings, working distances, spot sizes, and magnifications, were adjusted to capture distinct morphological details of the nanoparticles as reported in [36].

### 4.5. Scavenging Free Radical Activity of Salvia Extract and SO-AgNPs

The free radical scavenging activity of the extracts and SO-AgNPs was evaluated using both the DPPH^•^ and ABTS^+•^ assays, with slight modifications. For the DPPH^•^ assay, a 60 μM solution of DPPH^•^ in ethanol was prepared, and 1 mL of this solution was mixed with 100 μL of extracts or SO-AgNPs at concentrations ranging from 0.05 to 1 mg/mL. After 15 min incubation, the absorbance was measured at 517 nm using a UV/Vis spectrophotometer, as per the protocol given in Moussa et al. [17], and the antioxidant activity was expressed as a percentage of inhibition using Equation (1). Similarly, for the ABTS^+•^ assay, a solution of 7 mM ABTS combined with 2.45 mM potassium persulfate in ethanol was incubated for 24 h and then diluted to an absorbance of 0.7 at 734 nm. Subsequently, 1425 μL of this solution was mixed with 50 μL of samples and incubated for 6 min at room temperature before measuring the absorbance. Both assays expressed outcomes in terms of IC_50_ values, indicating the concentration required to inhibit 50% of the radicals, with lower IC_50_ values signifying higher antioxidant activity. Equation (1) was used for both assays to calculate the percentage of free radical inhibition:

(1) $$Free\;radical\;inhibition\;\left(\%\right)=\frac{A_{blanc}-A_{Sample}}{A_{Control}}*100$$

where $A_{Sample}$ is the absorbance of free radical solution (ABTS^+•^ or DPPH^•^) + sample extract at the required time, $A_{blanc}$ is the absorbance of free radical solution (ABTS^+•^ or DPPH^•^) + extraction solvent, and $A_{Control}$ is the absorbance of the working free radical solution.

### 4.6. Antimicrobial Activity of SO-AgNPs and Optimal Extract from S. officinalis L. Against Reference Strains of Bacteria

The antimicrobial activity of biosynthesized SO-AgNPs and optimal extract were evaluated against *Staphylococcus aureus* (ATCC 6538) (Food born), *Escherichia coli* (ATCC 25922), *Staphylococcus epidermidis* (clinical sample), *Bacillus subtilis* (ATCC 6633), *Methicillin-resistant staphylococcus aureus* (MRSA) (ATCC 43300), *Acinetobacter baumannii* (610), *Pseudomonas aeruginosa* (ATCC 6633), *Enterococcus faecalis* (ATCC 29212), and *Klebsiella pneumoniae* (laboratory stains) by the well diffusion method [24]. The pure cultures of bacteria were sub-cultured on a nutrient agar medium for 16–18 h. The suspension was standardized by adjusting the optical density to 0.08 to 0.1 at 620 nm (Optima, SP-3000nano, 5T5701-143132-00, Horiba Ltd., Kyoto, Japan). The wells of 6 mm diameter were made on Luria–Bertani (LA) agar plates then each strain was swabbed uniformly onto the individual LA plates using sterile cotton swabs. In total, 50 µL of SO-AgNPs (1 mg/mL in 5% of DMSO), optimal extract (50 mg/mL in 30% of DMSO), and negative controls (5 and 30% of DMSO) were poured onto each well on all plates. After incubation at 37 °C for 24 h, the zones of clearance around the wells after the incubation period confirmed the antimicrobial activity, and the average diameter of the inhibition zone (mm) was taken for evaluating the antimicrobial activity of the optimal extract of *S. officinalis* L. and SO-AgNPs. The minimal inhibitory concentration (MIC) was assessed using the serial microdilution broth susceptibility assay and the tetrazolium red salt (TTC) colorimetric assay, with slight modifications based on the method described in Hamid et al. [38]. In this assay, viable microorganisms interact with TTC, resulting in a color change from colorless to reddish-pink. The MIC was determined as the lowest concentration in the wells that did not exhibit this color change, indicating the inhibition of microbial growth.

### 4.7. Antimicrobial Activity of SO-AgNPs and Optimal Extract from S. officinalis L. Against Strains Bacteria of Industrial Effluent

Effluent samples from the FAIZ LAIT industrial waste manufacturing facility in the Corso Industrial Zone, Boumerdes, were collected. To evaluate the impacts of *S. officinalis* L. extract, and SO-AgNPs on the effluent, a thorough evaluation was conducted. Specific culture media were prepared, sterilized, and inoculated with bacteria from the effluent, followed by incubation and absorbance measurement of bacterial suspensions at 620 nm, yielding a 0.5 McFarland. The antibacterial effects of samples were assessed using the well diffusion method on Mueller-Hinton agar, showing inhibition zones after 24 h incubation at 37 °C [38]. Furthermore, the MICs of both samples were determined using the broth dilution method. [39,40,41,42,43]. A total of 50 µL of bacterial cultures adjusted to an absorbance of 0.07 nm in LB broth was added to serial dilutions of the test substances with concentrations ranging from 2 to 0.0625 mg/mL for the extract and from 1 to 0.03125 mg/mL for the SO-AgNPs. The cultures were incubated at 37 °C for 24 h, and the MIC was identified as the lowest concentration at which no visible microbial growth was observe [44,45,46,47,48,49,50,51,52,53,54].

### 4.8. Statistical Analyses

The GraphPad (9.5.0 (730)) and Origin software (2019b, 9.6.5.169) were utilized for data analysis, enabling both the construction of graphs and thorough statistical evaluation, which contributed to the study’s depth and reliability. All experiments were conducted in triplicate, with results presented as mean ± SD.

## 5. Conclusions

The study demonstrates that the green synthesis of SO-AgNPs is not only an environmentally sustainable method but also yields nanoparticles with substantial antioxidant and antibacterial properties. The optimized synthesis conditions resulted in SO-AgNPs that effectively scavenged ABTS^+•^ and DPPH^•^ radicals, with IC_50_ values of 0.305 and 0.185 mg/mL, respectively, indicating strong antioxidant activity. Additionally, both the *Salvia* extract and the synthesized SO-AgNPs showed significant antibacterial activity against many bacterial strains (MIC of 0.25 for SO-AgNPs and 3.12 and 6.25 mg/mL for *Salvia* extract, respectively), including those isolated from industrial effluents (MIC of 0.125 and 0.5 mg/mL for SO-AgNPs and *salvia* extract, respectively), highlighting their potential for biomedical and environmental applications. These findings underscore the dual functionality and promising applicability of *S. officinalis* L. extract and its derived SO-AgNPs, advocating for their further exploration and utilization in health and environmental fields. The notable antioxidant and antibacterial efficacy of these green synthesized nanoparticles points to their utility in developing new therapeutic agents and eco-friendly antimicrobial materials. Future research could explore the mechanisms underlying these bioactivities and evaluate the long-term stability and safety of SO-AgNPs in real-world applications. Overall, the integration of plant extracts in nanoparticle synthesis presents a valuable strategy for advancing nanotechnology in an eco-conscious manner.

## Figures and Tables

**Table 1:** Particle size and Zeta potential analysis of SO-AgNPs using Horiba SZ-100 DLS instrument.

	Peak No.	S.P. Area Ratio	Mean (nm)	S.D. (nm)	Mode (nm)	Z-Average (nm)	PI
Particle size (nm)	1	0.23	29.6	2.2	29.5	111.7	0.320
	2	0.77	130.5	12.9	129		
	Total	1	107.1	44.1	129		
Zeta potential	Zeta Potential mean (mV)			Electrophoretic mobility mean (cm^2^/Vs)			
	−21.2			−0.000164			

**Table 2:** Antimicrobial activity of *S. officinalis* L. leaves and SO-AgNPs against pathogenic bacteria strains using well diffusion method.

Pathogenic Bacteria	Type Strain	*S. officinalis* L. Extract	*S. officinalis* L. Extract (50 mg/mL)	SO-AgNPs	SO-AgNPs
Strains		MIC (mg/mL)		MIC (mg/mL)	(1 mg/mL)
*Staphylococcus aureus*	(ATCC 6538) (Food born)	N/A	Negative	N/A	14.67 ± 0.57
*Escherichia coli*	(ATCC 25922)	6.25	Negative		15
				0.25	
*Staphylococcus epidermidis*	clinical sample	3.12	Negative		14.67 ± 0.57
				0.25	
*Bacillus subtilis*	ATCC 6633	N/A	Negative		15
				N/A	
*Bacillus cereus*	Laboratory stains	6.25	18.33 ± 1.15	0.25	14.67 ± 0.57
*Methicillin-resistant*	ATCC 43300	6.25	16.67 ± 1.52	0.25	14.67 ± 1.52
*staphylococcus aureus* (MRSA)					
*Acinetobacter baumannii*	610	3.12	Negative	0.25	15
*Pseudomonas aeruginosa*	ATCC 6633	3.12	15	0.25	Negative
*Enterococcus faecalis*	ATCC 29212	N/A	11.33 ± 0.57	N/A	13.33 ± 1.15
*Klebsiella pneumoniae*	Laboratory stains	N/A	Negative	N/A	Negative
*Salmonella* spp.	Laboratory stains	N/A	15.67 ± 2.88	N/A	15 ± 1

SO-AgNPs: Silver nanoparticles; MIC: Minimal inhibitory concentration; all the diameters of the inhibition zone and MIC were measured in triplicate; N/A: Not Assessed.

**Table 3:** Antimicrobial activity of AgNPs synthesized using different plant extracts.

Plant Extract	Metallic Nanoparticles	Microbial Strains	Antimicrobial Activity (Inhibition Zone (mm)	References
*Symphyti radix (Boraginaceae; Sym. Radix)*	AgNPs	*S. aureus*	5.20 + 0.05	[28]
		*S. epidermidis*	5.40 + 0.10	
		*E. coli*	4.10 + 0.20	
		*K. pneumoniae*	3.10 + 0.05	
		*P. aeruginosa*	4.60 + 0.15	
		*B. cereus*	4.70 + 0.01	
*Salvia officinalis L.*	AgNPs	*S. aureus*	15.25 ± 0.25	[29]
		*S. epidermidis*	16.75 ± 1.75	
		*E. coli*	10.75 ± 0.75	
		*P. aeruginosa*	9.5 ± 0	
*Salvia officinalis L.*	AgNPs	*S. aureus*	24.4 ± 0.05	[30]
		*B. cereus*	24.0 ± 0.10	
		*B. subtilis*	18.8 ± 0.18	
		*E. coli*	22.4 ± 0.03	
		*P. aeruginosa*	20.9 ± 0.27	
		*K. pneumoniae*	23.0 ± 0.14	
*Eucalyptus globulus L.*	AgNPs	*S. aureus*	20.0 ± 0.10	[30]
		*B. cereus*	20.4 ± 0.08	
		*B. subtilis*	20.0 ± 0.20	
		*E. coli*	19.0 ± 0.01	
		*P. aeruginosa*	20.1 ± 0.03	
		*K. pneumoniae*	21.5 ± 0.01	
*Aloe fleurentinorum*	AgNPs	*S. Aureus*	10	[31]
		*B. Subtilis*	7	
		*E. Coli*	18	
*Hyssopus officinalis (Hyssopus)*	AgNPs	*S. aureus*	14.50 ± 0.15	[32]
		*S. epidermidis*	16.50 ± 0.10	
		*E. coli*	12.50 ± 0.10	
		*K. pneumoniae*	10.40 ± 0.10	
		*P. aeruginosa*	10.00 ± 0.90	
		*B. cereus*	9.50 ± 0.10	
		*E. faecalis*	10.00 ± 0.50	
*Calendula officinalis*	AgNPs	*S. aureus*	15.10 ± 0.10	[32]
		*S. epidermidis*	12.50 ± 0.10	
		*E. coli*	10.50 ± 0.35	
		*K. pneumoniae*	10.00 ± 0.10	
		*P. aeruginosa*	10.00 ± 0.10	
		*B. cereus*	11.80 ± 0.55	
		*E. faecalis*	10.40 ± 0.50	

## Data Availability

The data is available in the article.
